# Peak Running, Mechanical, and Physiological Demands of Professional Men’s Field Hockey Matches

**DOI:** 10.5114/jhk/161551

**Published:** 2023-04-20

**Authors:** Liya Lin, Xinyi Ji, Li Zhang, Haiqin Weng, Xinyi Wang

**Affiliations:** 1 Sports Training Research Center, Guangdong Institute of Sport Science, Guangzhou, China.; 2 APAC Team, Catapult Sports, Melbourne, Australia.; 3 Coaching Team, Guangdong Men’s Field Hockey Team, Guangzhou, China.

**Keywords:** GPS, activity profile, heart rate, team sports, worst case scenario

## Abstract

The aim of this study was to investigate the peak running, mechanical, and physiological demands of players of different positions in professional men's field hockey matches. Eighteen professional male field hockey players participated in the study, and data were collected in eleven official matches. Players wore GPS units (Vector S7, Catapult Sports) and heart rate (HR) monitors (Polar H1, Polar Electros) to collect physical and physiological data. Physical and physiological output of forwards, midfielders, and defenders in full matches and during 1-min peak periods was analysed. For all metrics and positions, the values identified for the 1-min peak periods were greater than the average values of match play (p < 0.05). In terms of 1-min peak period Player Load, all three positions were significantly different from each other. Forwards achieved the highest Player Load per minute, while defenders the lowest. The distance per minute, high-speed distance per minute, and the relative average heart rate of defenders were significantly lower than of midfielders and forwards (p < 0.05). The current study revealed the peak running, mechanical, and physiological demands of professional men’s field hockey matches. It is recommended when prescribing training programmes, to consider not only match average demands, but also peak demands. Forwards and midfielders displayed similar peak demands, while defenders had the lowest demands in all metrics except the number of accelerations and decelerations per minute. Player Load per minute can be used to identify the differences in peak mechanical demands between forwards and midfielders.

## Introduction

Using wearable technologies to monitor performance in team invasion sports such as football, field hockey, and rugby has become increasingly common in order to understand match physical demands, mitigate injury risks, and quantify training loads (Torres-Ronda et al., 2022). Distance and speed-related information can be reported using the Global Positioning System (GPS), and GPS units in athletes’ tracking are usually integrated with other sensors such as accelerometers, magnetometers, and gyroscopes to quantify sharp, short, and explosive movements such as change of directions, jumps, and movement patterns (Chambers et al., 2015; Lutz et al., 2019; Szigeti et al., 2021). As a fast-paced and intermittent sport, the physical condition of field hockey players is instrumental to the individual’s and the team’s match performance (Harry and Booysen, 2020; McGuinness et al., 2019; Morencos et al., 2018; Sarkar et al., 2019).

In recent studies on the physical demands of field hockey matches, the most frequently used metrics are total distance covered and distance covered in each speed zone. There are also numerous studies that include the numbers of accelerations and decelerations, heart rate metrics (Lim et al., 2021), and Player Load (McMahon and Kennedy, 2019; White and MacFarlane, 2013). Most of the studies utilise GPS units to collect data and analyse the activity profiles of match play, including match distance, match speed, and match duration profiles (Lim et al., 2021). More specifically, the comparisons have been made among different playing positions (Ihsan et al., 2021; McGuinness et al., 2019, 2020; Morencos et al., 2018), different playing rules (McMahon and Kennedy, 2019), and different competition levels (Vinson et al., 2018). In recent years, there have been trends of analysing not only the match average demands, but also the peak intensities of match demands (Cunniffe et al., 2021; Delves et al., 2021; Dewar and Clarke, 2021; Forouhaneh, 2020; McGuinness et al., 2020). However, those studies primarily examined the peak running intensities (i.e., running distance and high speed running distance), and rarely reported mechanical intensities (i.e., the number of accelerations and decelerations, Player Load, etc.). None of the peak intensity studies in field hockey reported physiological data (i.e., heart rate). Previous research suggested acceleration and deceleration metrics should be considered as match play fatigue indicators since they are metabolically challenging (Morencos et al., 2018). Individual responses to a certain external stimulus can be different between athletes and can be better indicated by internal load metrics such as the heart rate (Tuft and Kavaliauskas, 2021). Previous studies have shown that the heart rate can be used to determine the level of intensity and physiological demands in intermittent sports (Esposito et al., 2004). Establishing mechanical demands and physiological demands, along with running demands, will give practitioners a more comprehensive picture of the overall match demands, so that they can develop specific training drills which prepare athletes to cope with various types of maximal intensity scenarios in match play.

Based on the aforementioned progress and gaps in the field hockey match peak demands research, the aim of the current study was to investigate the peak running, mechanical, and physiological demands of players of different positions in professional men's field hockey matches.

## Methods

### Participants

Eighteen professional male field hockey players (25.6 ± 2.5 years, 171.8 ± 5.2 cm, 70.1 ± 7.5 kg) participated in the study, including 7 forwards, 6 midfielders, and 5 defenders. Participants worked full-time as professional athletes and trained at least 5 days per week, 2 sessions per day throughout the year. The data were collected during the 2021 National Games of China, where 11 official matches were played. The series of matches consisted of three phases, and each phase had 4, 3, and 4 matches respectively. Example of the match schedule in one phase can be found in Table 1. The team became the National Games champion and represented the highest national competition level. Prior to the data collection, all athletes were verbally informed of the purpose, procedures, risks, and benefits of the research, and written informed consent forms were signed. The research was approved by the Ethics Committee of the Guangdong Institute of Sport Science.

**Table 1:** Example of the match structure during one competition phase.

Day	Type
1	Match
2	Recovery
3	Training
4	Match
5	Match
6	Recovery
7	Training
8	Match

### Measures

The physical data were collected using GPS technology integrated with inertial sensors (Vector S7, Catapult Sports, Melbourne, Australia, Firmware Version 8.1). The GPS sampling frequency was 10 Hz, and the inertial sensor sampling frequency was 100 Hz. The physiological data were collected using heart rate monitors (Polar H1 chest strap, Polar Electro Oy, Kempele, Finland). The validity and reliability of both devices have been reported acceptable (Clavel et al., 2022; Crang et al., 2022; Schaffarczyk et al., 2022). Both physical data and physiological data were processed in OpenField (Catapult Sports, Melbourne, Australia, Version 3.3.1).

The physical demand metrics reported included:

- Distance per minute (m·min^-1^);
- High-speed running distance per minute (m·min^-1^), threshold set at > 15 km·h^-1^, which was utilised in previous studies (Ihsan et al., 2021; McMahon and Kennedy, 2019; White and MacFarlane, 2013)
- Player Load per minute (au), derived from the accelerometer in the GPS unit, sampling at 100 Hz, being the sum of the accelerations across all axes of the internal tri-axial accelerometer during movement. It takes into account the instantaneous rate of change of acceleration and divides it by a scaling factor (divided by 100). Player Load reports the total external mechanical stress accumulated (Lutz et al., 2019);
- Number of accelerations and decelerations per minute (n·min^-1^), threshold set at > 2 m·s^-2^;
- The physiological demand metric reported included:
- Relative average heart rate (%), the individualised average heart rate calculated from the maximal heart rate.

The individual maximal heart rate was collected from all the Yo-Yo IR2 tests, training and matches in the 12-month period prior to the tournament. Research shows that the maximal heart rate collected from Yo-Yo IR2 tests has no difference from the maximal heart rate collected from conventional laboratory treadmill incremental tests to exhaustion (Bradley et al., 2011).

Considering the nature of field hockey matches, where unlimited substitutions are allowed, all the running and mechanical variables were normalised by the actual on-pitch duration of players to reflect the intensity of match play.

### Design and Procedures

The data were collected from 18 professional male field hockey players in 11 official matches in the National Games Series. Players were categorised into 3 positions: forwards, midfielders, and defenders. Overall, there were 169 observations included in the dataset (64 for forwards, 62 for midfielders, and 43 for defenders).

Players wore their own GPS unit, GPS vest, and heart rate straps throughout the tournament to ensure consistency. Prior to each match, GPS units were turned on at least fifteen minutes before the warm-up and the heart rate sensors were moisturised as per the manufacturers’ instructions to ensure optimal data quality. The average number of satellites connected was 14.99 ± 1.66, and the average horizontal dilution of precision (HDOP) was 0.73 ± 0.08, which can be considered good satellite signal quality (Malone et al., 2017). Physical and physiological data were processed and trimmed according to the actual playing time of players: the between-quarter breaks, off-pitch time, and video review time were excluded from the analysis.

The time-series data were exported to customized Excel spreadsheets (Microsoft, Redmond, USA, Version 16) to calculate the moving averages of players. The moving averages method was chosen over predefined periods because the predefined periods might underestimate peak running demands by up to 25% (Varley et al., 2012). Therefore, we used moving averages to provide a more accurate representation of the peak intensity. It was reported that physical demands increase as the duration of the moving average decreases (McGuinness et al., 2020). The 1-min moving window was chosen to calculate the peak demands, because of the fast-paced nature of field hockey matches and the frequency that the matches were interrupted and stopped. In the present study, continuous play duration in the matches was mostly less than 2 minutes. Therefore, we chose to use a 1-min period to reflect the peak intensity during match play. For distance per minute, high-speed running distance per minute, Player Load per minute, and relative average heart rate, the moving window was 60 s, and the moving interval was 1 s (i.e., 0–60 s, 1–61 s, 2–62 s, 3–63 s), to determine the peak 1-min period of each metric. For the number of accelerations and decelerations per minute, the moving window was 60 s, and the moving interval was 10 s (i.e., 0–60 s, 10–70 s, 20–80 s, 30–90 s) to determine the peak 1-min period of accelerations and decelerations. The reason to use a 10-s interval was that the movement of acceleration and deceleration is not instantaneous, and one action can sustain a few seconds.

### Statistical Analysis

Data are expressed as the mean ± standard deviation. The repeated measures ANOVA was used to determine the differences across positions. In the event of a significant difference, the Bonferroni post hoc test was used. Statistical significance was set at *p* < 0.05. All statistical analyses were conducted using IBM SPSS Statistics (IBM Corp., Armonk, USA, Version 27).

## Results

**Figure 1:**
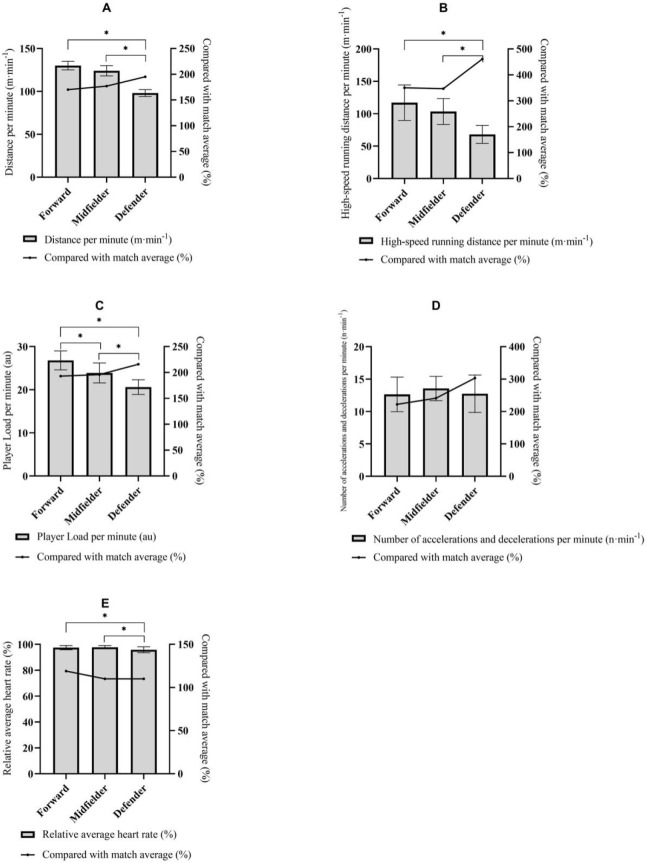
Peak 1-min demands of distance per minute (A), high-speed running distance per minute (B), Player Load per minute (C), number of accelerations and decelerations per minute (D), and relative heart rate (E) of different positions, with annotation for statistically significant differences and percentages relative to match averages. * *indicates significant differences between different position groups (p < 0.05)*

The match average demands and peak 1-min demands of running, mechanical, and physiological metrics of different positions are presented in Table 2 and Figure 1.

For all metrics and positions, the values identified for the 1-min peak periods were greater than the average values of match play (*p* < 0.05). In terms of peak 1-min period Player Load, midfielders, forwards, and defenders were significantly different from each other. Forwards achieved the highest Player Load per minute, while defenders the lowest. The distance per minute, high-speed distance per minute, and the relative average heart rate of defenders were significantly lower than of midfielders and forwards (*p* < 0.05).

**Table 2:** Match average demands and peak 1-min demands of running, mechanical, and physiological intensities of different positions.

		Midfielders	Forwards	Defenders
Match Average	Distance per minute (m·min^-1^)	124 ± 6	130 ± 5	98 ± 4
	High-speed running distance per minute (m·min^-1^)	29.8 ± 4.7	33.5 ± 3.9	14.8 ± 2.5
	Player Load per minute(au)	12.2 ± 0.7	13.9 ± 0.6	9.5 ± 0.6
	Number of accelerations and decelerations per minute (n·min^-1^)	5.63 ± 0.5	5.68 ± 0.51	4.2 ± 0.41
	Relative average heart rate (%)	88.9 ± 2.7	81.6 ± 2.4	86.8 ± 2.3
Peak 1-min Periods	Distance per minute (m·min^-1^)	220 ± 15^a^	221 ± 11 ^a^	192 ± 9 ^a^
	Compared with match average(%)	177%	170%	195%
	High-speed running distance per minute (m·min^-1^)	103.4 ± 20 ^a^	117 ± 27.4 ^a^	68.1 ± 13.8 ^a^
	Compared with match average(%)	347%	350%	461%
	Player Load per minute(au)	23.9 ± 2.3 ^a^	26.8 ± 2.2 ^a^	20.6 ± 1.7 ^a^
	Compared with match average(%)	196%	193%	216%
	Number of accelerations and decelerations per minute (n·min^-1^)	13.55 ± 1.88 ^a^	12.64 ± 2.66 ^a^	12.74 ± 2.89 ^a^
	Compared with match average(%)	241%	222%	303%
	Relative average heart rate (%)	97.8 ± 1.3 ^a^	97.5 ± 1.6 ^a^	95.8 ± 2.3 ^a^
	Compared with match average(%)	110%	119%	110%

a indicates significant differences between match average demands and peak 1-min demands (p < 0.05)

## Discussion

The current study investigated peak physical and physiological demands in professional men’s field hockey players of different positions. To the authors’ knowledge, it has been the first study to outline not only peak running and high-speed running intensities, but also peak mechanical and physiological intensities of professional men’s field hockey matches.

Referencing the match intensity when prescribing training programmes has become common sense amongst modern field hockey coaches. Coaches would endeavour to replicate the match's physical demands in training. However, coaches often use match average data as a reference, which might underestimate peak match demands and underprepare players for real match play (McGuinness et al., 2020). Peak demands analysis has been utilised in football and rugby studies for more than a decade. However, it was only recently investigated in field hockey (Cunniffe et al., 2021; Delves et al., 2021; Forouhaneh, 2020; McGuinness et al., 2020). This type of analysis in field hockey primarily investigated peak running intensity, it was less focused on peak high-speed running intensity, and no research has investigated the amount of accelerations and decelerations in the most intensive 1-min period. There were no studies on peak Player Load per minute, and heart rate data during peak periods.

The results showed that forwards and midfielders had similar peak demands in terms of distance per minute (221 ± 11 m and 220 ± 15 m, respectively), and were significantly higher than defenders (192 ± 9 m). The pattern was consistent with the results from previous studies (Delves et al., 2021; Dewar and Clarke, 2021; Forouhaneh, 2020). However, the distance per minute values of the current study are similar to what was reported by Delves et al. (2021), but greater than what was reported by Dewar and Clarke (2021), and Forouhaneh (2020). This might be due to the different athletes’ levels of those studies. Participants in the previous studies were reported to be sub-elite athletes, while players in the current study were full-time professional athletes. It was suggested that the peak relative total distance was impacted by the competition level (Cunniffe et al., 2021). From the positional difference perspective, the current study is consistent with previous research that the peak high-speed running demands of forwards and midfielders are similar and are both higher than those of defenders. Forwards achieved 117 ± 27.4 m of high-speed running in the peak 1-min period, and midfielders achieved 103.4 ± 20 m, which were all higher than in defenders (68.1 ± 13.8 m). These are similar to the findings of McGuinness et al. (2020). However, the defender’s peak high-speed running demands were lower than what was reported by McGuiness et al. (2020), although the previous study was conducted with female athletes, and the high-speed threshold (16 km·h^-1^) was also defined higher than in the current study. The potential reason could be that the defenders of the current study were full backs in the matches and rarely shared the responsibilities of attacking or aggressively pushing forward in the defense-to-attack transitions. Therefore, it was expected that defenders in the current research did not generate much high-speed running even when compared with defenders from other teams who might be responsible for defense-to-attack transitions.

The peak 1-min Player Load demands were significantly different across all positions. Forwards presented the highest demands, while defenders the lowest. As previously reported by Lutz et al. (2019), Player Load reports the total external mechanical stress accumulated. This may indicate that Player Load per minute can be used as a sensitive indicator to identify the differences in peak mechanical demands between forwards and midfielders. There was no significant difference in terms of the number of accelerations and decelerations in the peak 1-min period across the three positions. Delves et al. (2021) reported the acceleration demands in the peak 1-min period using instantaneous acceleration magnitude (m·s^-2^), as opposed to the number of accelerations and decelerations (n). They showed that male defenders performed at greater intensities when compared with midfielders (Delves et al., 2021). Although the current study does not report significantly higher peak accelerations and decelerations in defenders when compared with forwards and midfielders, it also does not indicate that the peak accelerations and decelerations of defenders were significantly lower than in the other positions, unlike other physical and physiological demands. These findings reflect the role and responsibilities of defenders which are marking the opponent players behind the play, and reactively but quickly responding to the opponent players’ movements in order to intercept or interfere with the opponents’ play. The nature of the responsibilities of defenders means that these plays are conducted in relatively smaller areas, without too much space for free running (Delves et al., 2021; Polglaze et al., 2015). On the contrary, forwards and midfielders need to cover a greater area on the pitch to create space for attacking, hence, the running demands are higher.

The relative average heart rate of defenders during the peak 1-min period was 95.8 ± 2.3%, which is significantly lower than in forwards and midfielders (97.5 ± 1.6% and 97.8 ± 1.3%, respectively). This could be due to the significantly lower external stimulus that defenders were exposed to. When looking at the respective peak intensity periods, defenders still had significantly lower demands in both physical and physiological intensities, compared to midfielders and forwards. Both peak running and physiological demands were similar for midfielders and forwards, but the positional difference could be revealed by Player Load per minute.

For all metrics and all positions, the 1-min peak period demands were significantly higher than match average intensities. These findings suggest that there is a huge gap between match average intensities and match peak intensities, and it is far from adequate to design training programmes based on match average intensity benchmarks, which could potentially underprepare players. The values reported in the current study can be used as reference points for peak match demands in professional men’s field hockey, and help coaches and practitioners in prescribing training drills that can expose players to appropriate peak physical and physiological demands.

When interpreting the results of the current study, the context of limitations should be considered. The data collected were only from one team during one competition. Therefore, our findings might not be transferrable to teams in different competition levels, using different formations, and with different playing styles. In addition, the team in the current study was the strongest team in the competition, the results of the 11 matches were 9 wins and 2 ties, thus, it was challenging to investigate the effects of opponent levels and match outcomes. The lack of tactical contexts (i.e., possession status, specific match events, rotations, etc.) also contributed to the limited analysis of the datasets. It is recommended that in future studies of peak demands in professional men’s field hockey matches, physical and physiological data should be collected from multiple teams, in multiple competitions, and integrate with tactical contexts to gain a better understanding of the match demands.

## Conclusions

Peak 1-min period intensities are higher than match average intensities by a large margin. When prescribing training programmes, coaches are encouraged to consider not only match average demands, but also peak demands.

Differences between average and peak demands are not proportional in all metrics, high-speed running per minute and the number of accelerations and decelerations per minute showed the largest difference between game average demands and the peak 1-min period.

In terms of positional differences, forwards and midfielders displayed similar peak demands, while defenders had the lowest demands in all metrics except the number of accelerations and decelerations per minute. Player Load per minute might be used to distinguish between the mechanical load demands of forwards and midfielders.

## Author Contributions

Conceptualization: L.L., X.J. and X.W.; methodology: L.L. and X.J.; software: X.J., H.W. and X.W.; validation: L.L., X.J. and L.Z.; formal analysis: L.L., X.J. and L.Z.; investigation: L.L., X.J. and L.Z.; resources: L.L., L.Z. and H.W.; data curation: X.J.; writing—original draft preparation: L.L. and X.J.; writing—review & editing: L.L., X.J. and L.Z.; visualization: X.J.; supervision: L.L.; project administration: L.L. and L.Z.; funding acquisition: L.L. and L.Z. All authors have read and agreed to the published version of the manuscript.

## ORCID iD

Liya Lin: 0000-0001-6071-7757

Xinyi Ji: 0000-0002-1587-7433
